# Nicotine, Humectants, and Tobacco-Specific Nitrosamines (TSNAs) in IQOS Heated Tobacco Products (HTPs): A Cross-Country Study

**DOI:** 10.3390/toxics12030180

**Published:** 2024-02-27

**Authors:** Noel J. Leigh, Michelle K. Page, Denisha L. Robinson, Scott D. Heldwein, Richard J. O’Connor, Maciej L. Goniewicz

**Affiliations:** 1 Department of Health Behavior, Roswell Park Comprehensive Cancer Center, Buffalo, NY 14263, USA; noel.leigh@roswellpark.org (N.J.L.); richard.oconnor@roswellpark.org (R.J.O.); 2 Department of Cell Stress Biology, Roswell Park Comprehensive Cancer Center, Buffalo, NY 14263, USA; denisha.robinson@roswellpark.org

**Keywords:** nicotine, humectants, TSNA, heated tobacco products, heat-not-burn, IQOS

## Abstract

Heated Tobacco Products (HTPs) purport to reduce exposure to tobacco-related toxicants compared to combustible cigarettes. This cross-sectional study examined the content of nicotine, two humectants (propylene glycol (PG) and vegetable glycerin (VG)), and four tobacco-specific nitrosamines (TSNAs: NNN, NNK, NAT, and NAB) in the tobacco filler of a popular HTP brand (IQOS). Non-menthol and menthol IQOS sticks were purchased from nine countries between 2017 and 2020 and were classified into two versions (“Bold” and “Light”) using Philip Morris’s flavor descriptors. The average nicotine concentration was 4.7 ± 0.5 mg/stick, and the highest nicotine concentration was found in products from Japan (5.1 ± 0.2 mg/stick). VG was the dominant humectant found in all sticks, with an average concentration of (31.5 ± 2.3 mg/stick). NNN, NNK, and NAT were substantially higher in the “Bold” sticks than the “Light” sticks. Significant differences between countries for TSNAs were also observed: the NAT and NAB contents were the highest in the “Light” products from Canada (192.5 ± 24.1 and 22.9 ± 1.0 ng/stick, respectively); the NNK concentration was the highest in the “Bold” products from Poland (64.8 ± 7.9 ng/stick); and the highest NNN concentrations were observed in the “Bold” products from South Africa (488.9 ± 26.7 ng/stick). As NNN and NNK are known human carcinogens, and as humectants like PG and VG can degrade into toxic carbonyl compounds upon heating, monitoring the concentration of these chemicals in HTPs is important for protecting users’ health and ensuring compliance with regulations.

## 1. Introduction

Heated Tobacco Products (HTPs) are a class of tobacco products that purport to deliver nicotine via inhalation while reducing exposure to tobacco-related toxicants compared to combustible cigarettes. Most HTPs use a similar design, consisting of a battery, a microprocessor, and a heating element in direct contact with a tobacco stick. These tobacco sticks look very similar to conventional tobacco cigarettes. Yet, they are typically much shorter and have a metal wrapper that encases the tobacco filler to prevent accidental or intentional combustible use [1]. HTPs release a nicotine-containing aerosol when heating these tobacco sticks up to 350 °C using an electronic heating element [2]. Since their introduction, HTPs have evolved, with changes to their battery life, the design of the heating element, and the constituents of tobacco sticks, including additives like humectants and flavorings [2].

Heated Tobacco Products (HTPs) were first introduced commercially in the 1980s. For a limited time, HTP brands like Accord and Eclipse were available on several markets [3]. However, due to their limited adoption by tobacco users, those products were eventually withdrawn by manufacturers. Contemporary brands of HTPs have been aggressively promoted by multiple international tobacco companies in the last decade. In 2014, the largest tobacco company, Phillip Morris International (PMI), launched its HTP brand, IQOS, in Italy and Japan, which were deemed “key markets” due to their large numbers of smokers, lower taxation, and weak tobacco control policy [4]. Since then, IQOS has become one of the most popular HTP brands on the market [5,6,7,8]. It is currently available in 61 countries [9], and tobacco sticks designed for use with IQOS (sold under the brand names Marlboro, HEETS, TEREA, or SENTIA) are available in various flavor options closely resembling the non-menthol and menthol flavored cigarettes available in each country. For example, in Canada, where Marlboro cigarettes are sold in two flavors (Red and Silver Labels), similar flavor types were also used in Marlboro IQOS sticks at the time of this study.

Tobacco products differ significantly in their chemical composition, including nicotine content and the presence of toxic ingredients. Various additives are used during tobacco curing and tobacco product manufacturing processes to increase nicotine delivery to users and enhance the palatability of the product. Those additives include humectants such as vegetable glycerin (VG) and propylene glycol (PG) that carry nicotine in the aerosols generated from HTPs. These humectants, when heated, may cause potential harm to users due to the formation of carbonyls (e.g., formaldehyde, acetaldehyde, and acrolein), [10] a class of compounds that can be cytotoxic, mutagenic, and carcinogenic [11]. In addition, IQOS has been shown to induce oxidative DNA damage as significantly as cigarettes [12]. Furthermore, the flavor profile of tobacco products is highly affected by the growth location and type of tobacco, as well as the additives used during the curing and manufacturing processes [13]. Tobacco is produced in 130 countries worldwide [14], with China as the dominant cultivator [15]. Tobacco leaves can be flue-cured, air-cured, fire-cured, and sun-dried [16]. The method of curing results in various flavor profiles and affects the chemical composition of the tobacco, including cancer-causing tobacco-specific nitrosamines (TSNAs) [16]. For example, air-cured tobacco, which is primarily grown in Argentina, Brazil, Italy, Malawi, and the United States [17], has been found to contain significantly higher levels of 4-(methylnitrosamino)-1-(3-pyridyl)-1-butanone (NNK) as compared to flue-cured tobacco [16]. Among the TSNAs, N′-nitrosonornicotine (NNN) and NNK are classified as the most potent human carcinogens [18]. These TSNAs have been shown to cause lung, oral, tracheal, nasal, and liver cancers in animal models [19,20,21,22,23].

Few studies have examined the chemical composition of HTPs, and most of the published studies focusing on the physicochemical properties of tobacco used in HTP sticks come from the tobacco industry [24]. Additionally, independent studies have yet to examine the chemical content of sticks in any popular HTP brand worldwide. As the leading brands of HTPs are becoming widely available in many countries, cross-country studies are needed to better understand the global trends in these emerging products and monitor modifications to the HTPs that are already commercially available. This study examined the nicotine, humectants, and TSNA content in IQOS tobacco sticks from nine countries in various parts of the world (North America, Europe, Africa, and Asia).

## 2. Materials and Methods

### 2.1. Product Purchasing and Classification

**Figure 1:**
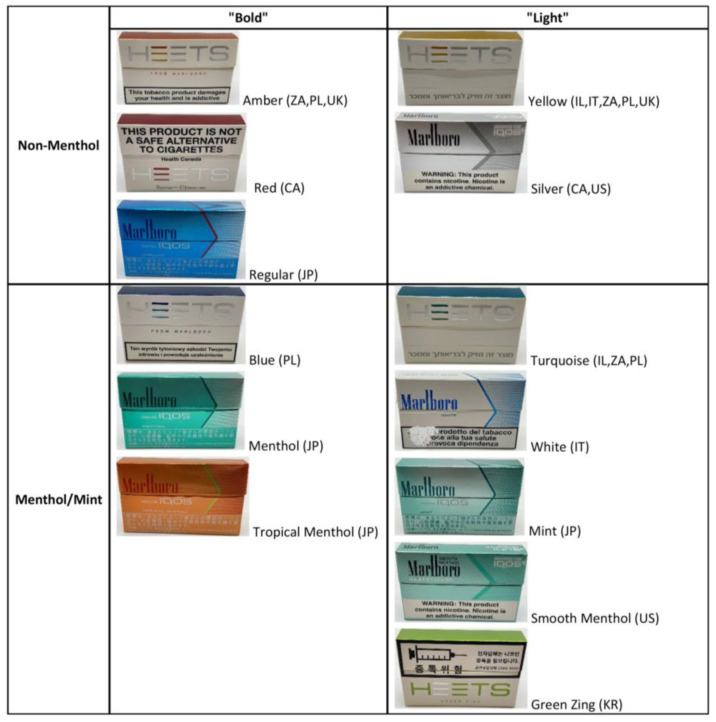
“Bold” vs. “Light” IQOS stick classification. Classification of “Bold” vs. “Light” was completed using PMI’s flavor descriptors on its country-specific websites; see Methods. For products purchased in multiple countries, only one example per product type is presented above. Small package differences were observed between countries; all sticks used in this study had flavor descriptors on their country-specific websites. Packaging for IQOS White was damaged during experiments. Key: Canada (CA), Israel (IL), Italy (IT), Japan (JP), Poland (PL), the Republic of Korea (KR), South Africa (ZA), the United Kingdom (UK), and the United States (US).

A convenient sample of IQOS sticks was purchased from convenience stores, IQOS flagship stores, and a IQOS online store from the following countries—Canada, Israel, Italy, Japan, Poland, the Republic of Korea, South Africa, the United Kingdom (UK), and the United States (US)—between 2017 and 2020. All the products were purchased in either non-menthol or menthol flavors with either “Bold” or “Light” profiles, as described in detail in Figure 1. Depending on the country, these products were sold under the HEETS or Marlboro brands. The classification of “Bold” vs. “Light” was completed using PMI’s flavor descriptors from its country-specific websites. For all sticks, the qualifier “intensity” was used to describe a particular “flavor” of a stick. If the percent intensity surpassed 50%, the product “flavor” was categorized as “Bold”. If the intensity was under 50%, the stick “flavor” was classified as “Light” (Figure 1). A single pack of each type of stick was purchased for all products. All products were conditioned at 22 ± 1 °C and 60 ± 2% RH for 48 h before testing following the Cooperative Centre for Scientific Research Relative to Tobacco (CORESTA) method N°21 [25]. Before analysis, a razor blade was used to remove the tobacco filler from a stick into a clean-weight boat, and the tobacco plug was weighed using an analytical balance.

### 2.2. Analysis of the Nicotine in the IQOS Sticks

The isolated tobacco filler from each stick was manually shredded using two razor blades until homogenous and extracted using CORESTA method N°62 [26]. One shredded tobacco plug (~0.3 g) was placed into a 250 mL Erlenmeyer flask with 7 mL of 5 N NaOH and allowed to stand for 15 min. Then, 50 mL of MTBE, with 0.4 mg/mL of internal standards, was added to the flask. The flask was capped and placed onto an orbital shaker for two hours, followed by a 15 min wait to allow the layers to separate. A 1 mL aliquot of the organic phase was transferred into an amber chromatography vial and analyzed using an Agilent 7890B gas chromatography system with a nitrogen phosphorus detector. The lowest limit of quantitation (LOQ) of the method was 0.6 mg/stick, with an average recovery of 102.7% and a calibration curve linearity of r2 ≥ 0.998.

### 2.3. Analysis of the Humectants in the IQOS Sticks

Two humectants, VG and PG, were extracted from the tobacco filler using a modified version of the CORESTA method N°92 [27]. Two tobacco plugs (~0.6 g) were weighed and placed into a 250 mL Erlenmeyer flask with 40 mL of methanol with 0.25 mg/mL of internal standards. The flask was capped and placed onto an orbital shaker for 2 h. A 1 mL aliquot was filtered using a 0.45 µm PTFE syringe filter into an amber chromatography vial and analyzed using an Agilent 7890B gas chromatography system and an Agilent 5977A mass spectrometer. The LOQ of the method was 0.2 and 0.02 mg/stick (VG and PG, respectively), with an average recovery of 99.9% and calibration curve linearities of r2 ≥ 0.99.

### 2.4. Analysis of the TSNAs in the IQOS Sticks

Each tobacco plug from each heat stick was removed, shredded, and extracted using CORESTA method N°72 [28]. Briefly, the tobacco was weighed and placed into a 250 mL Erlenmeyer flask with 30 mL of 100 mM ammonium acetate and 10 µg/mL internal standard solution. The flask was capped and put onto an orbital shaker for 40 min. A 1 mL aliquot was filtered using a 0.45 µm PTFE syringe filter into an amber chromatography vial and analyzed using a Waters I-Class liquid chromatography system and a Xevo TQ-S mass spectrometer. The following TSNAs were measured in this study: NNN, NNK, N’-nitrosoanatabine (NAT), and N’-nitrosoanabasine (NAB). NNN and NNK had a LOQ of 15.0 ng/stick, while NAT and NAB had a LOQ of 7.5 ng/stick. The average recoveries were between 98.6 and 103.5%, and the linearities of the calibration curves were r2 ≥ 0.998.

### 2.5. Statistical Analysis

**Figure 2:**
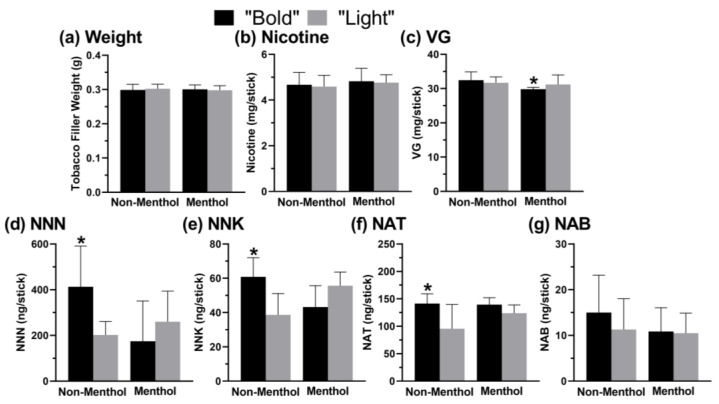
Chemical characteristics of “Bold” and “Light” IQOS sticks purchased in non-menthol and menthol flavors; see Figure 1 for classification. Mann–Whitney non-parametric *t*-tests were used to compare each study variable between “Bold” and “Light” for non-menthol and menthol sticks. Error bars show standard deviation. Number of product types per category: non-menthol “Bold” n = 5, non-menthol “Light” n = 7, menthol “Bold” n = 3, menthol “Light” n = 7. All products were extracted and analyzed at least three times. Key: (**a**) weight, (**b**) nicotine, (**c**) vegetable glycerin (VG), (**d**) N’-nitrosonornicotine (NNN), (**e**) 4-(methylnitrosamino)-1-(3-pyridyl)-1-butanone (NNK), (**f**) N’-nitrosoanatabine (NAT), and (**g**) N-nitrosoanabasine (NAB); * = *p* < 0.05 compared to “Light” within flavor (i.e., menthol or non-menthol); see Results for exact *p* values.

**Figure 3:**
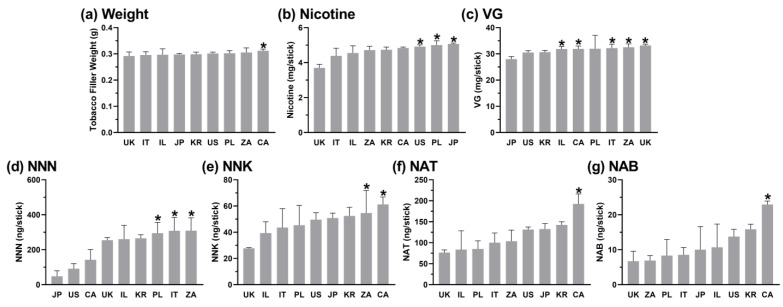
Chemical characteristics of “Light” IQOS sticks compared by country of purchase. Kruskal–Wallis non-parametric ANOVA with Dunn’s multiple-comparison tests was used to compare the mean rank of the country with the lowest concentration of each variable to the mean rank of each other country. Error bars show standard deviation. For comparisons of mean rank of each country with every other country, see Table S2. Number of “Light” products per country; CA n = 1, IL n = 2, IT n = 2, JP n = 1, PL n = 2, ZA n = 2, KR n = 1, UK n = 1, US n = 2; see Figure 1 legend for country abbreviations. All products were extracted and analyzed at least three times. * = statistically significant (*p* < 0.05) between the country with the lowest value and this country; see Results for exact *p* values. See Figure 2 legend for a key.

**Figure 4:**
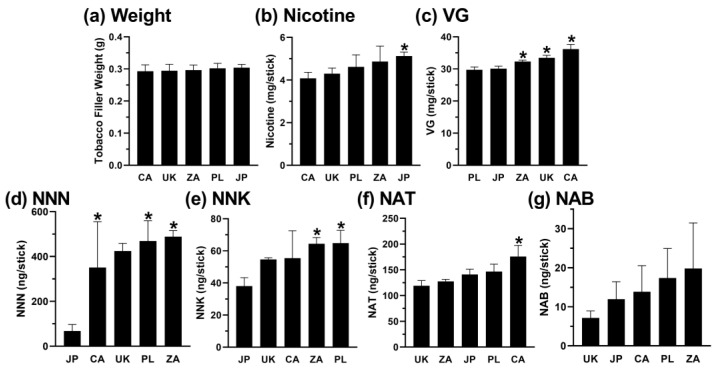
Chemical characteristics of “Bold” IQOS sticks compared by country of purchase. Kruskal–Wallis non-parametric ANOVA with Dunn’s multiple-comparison tests were used to compare the mean rank of the country with the lowest concentration of each variable to the mean rank of each other country. Error bars show standard deviation. For comparisons of the mean rank of each country with every other country, see Table S3. Number of “Bold” products per country; CA n = 1, JP n = 3, PL n = 2, ZA n = 1, UK n = 1; see Figure 1 legend for country abbreviations. All products were extracted and analyzed at least three times. * = statistically significant (*p* < 0.05) between the country with the lowest value and this country; see Results for exact *p* values. See Figure 2 legend for a key.

Statistical analysis was performed using GraphPad Prism version 9.5.1 (733). Mann–Whitney non-parametric *t*-tests were used to compare each study variable between “Bold” and “Light” for the non-menthol as well as the menthol sticks (Figure 2). Kruskal–Wallis non-parametric ANOVA and Dunn’s multiple-comparison tests were performed to compare each study variable between countries for the “Bold” and “Light” sticks (Figure 3 and Figure 4). The country with the lowest concentration for each variable is represented as the reference in these figures. The mean rank of each country per variable was also compared in Tables S2 and S3. For each IQOS product, we selected sticks randomly from a single pack, and three results were generated for each analytical method, as described above. All the results were presented as the mass of chemicals per stick.

## 3. Results

### 3.1. Weight of Tobacco Filler in the IQOS Sticks

The average tobacco plug weight for all the analyzed IQOS sticks was 0.30 ± 0.01 g. In the “Light” category, the lowest weight was observed in the sticks from the UK and the highest weight in the products from Canada (*p* = 0.028, H(8) = 14.2; Figure 3a). In the “Bold” category, the lowest weight was observed for the sticks from Canada, while the products from Japan had the highest weight (*p* > 0.05, H(4) = 3.5; Figure 4a).

### 3.2. Nicotine Content in the IQOS Sticks

Among all the sticks analyzed, the average nicotine concentration was 4.7 ± 0.5 mg/stick (16.0 ± 1.3 mg/g of tobacco). In the “Light” category, the lowest nicotine content was observed in the sticks from the UK and the highest nicotine content in products from Japan (*p* = 0.004, H(8) = 24.0; Figure 3b). When the “Light” category was compared by country, significant differences were also observed between the UK and the US and the UK and Poland (*p* = 0.017 and *p* = 0.007, respectively; Figure 3b). In the “Bold” category, the lowest nicotine content was observed in the sticks from Canada, and the highest nicotine content was found in the products from Japan (*p* = 0.001, H(4) = 15.3; Figure 4b).

### 3.3. Humectant Content in the IQOS Sticks

Vegetable glycerin (VG) was the dominant humectant found in all the analyzed sticks. Propylene glycol (PG) was detected in all the sticks examined in this study, and on average, the PG content was about 100× lower than the average VG content in the sticks. For this reason, we only present the results on the VG content in the sticks.

Among all the products analyzed in this study, the individual sticks contained, on average, 31.5 ± 2.3 mg/stick (104.1 ± 7.0 mg/g) of VG. Significant differences between the “Bold” and “Light” sticks were observed for the menthol but not the non-menthol sticks (*p* < 0.001, U = 324.5; Figure 2c). In the “Light” category, the lowest VG content was observed in the sticks from Japan and the highest VG content in products from the UK (*p* < 0.001, H(8) = 52.1; Figure 3c). When the “Light” category was compared by country, significant differences were also observed between Japan and Canada (*p* = 0.001), Japan and Israel, Japan and Italy, and Japan and South Africa (*p* < 0.001 for all others; Figure 3c). In the “Bold” category, the lowest VG content was observed in the sticks from Poland and the highest VG content in products from Canada (*p* < 0.001, H(4) = 52.3; Figure 4c). When the “Bold” category was compared by country, significant differences were also observed between Poland and South Africa and Poland and the UK (*p* = 0.002 and *p* < 0.001, respectively; Figure 4c).

### 3.4. TSNA Content in the IQOS Sticks

Among all the products analyzed, the average stick contained 255.7 ± 154.7 ng/stick (826.0 ± 494.1 ng/g tobacco) of NNN. When the “Bold” and “Light” categories were compared, significant differences in the NNN levels were observed for the non-menthol-flavored products (*p* = 0.001, U = 59.5; Figure 2d). In the “Light” category, the lowest NNN content was observed in the sticks from Japan and the highest in the products from South Africa (*p* = 0.013, H(8) = 29.8; Figure 3d). When the “Light” category was compared by country, significant differences in the NNN levels were also observed between Japan and Poland and Japan and Italy (*p* = 0.019 and *p* = 0.015, respectively; Figure 3d). In the “Bold” category, the lowest NNN content was observed in the sticks from Japan and the highest NNN content in the products from South Africa (*p* = 0.007, H(4) = 18.2; Figure 4d). When the “Bold” category was compared by country, significant differences in the NNN levels were also observed between Japan and Canada and Japan and Poland (*p* = 0.009 and *p* = 0.004, respectively; Figure 4d).

Among all the sticks, the average NNK concentration was 49.7 ± 13.7 ng/stick (161.1 ± 43.0 ng/g tobacco). When the “Bold” and “Light” categories were compared, significant differences were observed for the non-menthol-flavored products (*p* < 0.001, U = 33.0; Figure 2e). In the “Light” category, the lowest NNK content was observed in the sticks from the UK and the highest NNK content in the products from Canada (*p* = 0.002, H(8) = 18.9; Figure 3e). When the “Light” category was compared by country, significant differences in NNK levels were also observed between the UK and South Africa (*p* = 0.031; Figure 3e). In the “Bold” category, the lowest NNK content was observed in the sticks from Japan and the highest NNK content in the products from Poland (*p* = 0.002, H(4) = 15.0; Figure 4e). When the “Bold” category was compared by country, significant differences in the NNK levels were also observed between Japan and South Africa (*p* = 0.041; Figure 4e).

Among all the products analyzed, the average heat stick contained 127.4 ± 39.1 ng/stick (414.3 ± 127.8 ng/g) of NAT. When the “Bold” and “Light” categories were compared, significant differences were observed within the non-menthol-flavored products (*p* = 0.002, U = 63.5; Figure 2f). In the “Light” and “Bold” categories, the lowest NAT content was observed in the sticks from the UK and the highest NAT content in the products from Canada (*p* = 0.003 and *p* < 0.001, respectively; H(8) = 31.9 and H(4) = 18.1, respectively; Figure 3f and Figure 4f, respectively).

Among all the analyzed sticks, the average stick contained 12.5 ± 6.6 ng/stick (40.6 ± 21.1 ng/g tobacco) of NAB. In the “Light” category, the lowest NAB content was observed in the sticks from the UK and the highest NAB content in the products from Canada (*p* = 0.006, H(8) = 26.7; Figure 3g). In the “Bold” category, the lowest NAB content was observed in the sticks from the UK and the highest NAB content in the products from South Africa (*p* > 0.05, H(4) = 5.7; Figure 4g).

## 4. Discussion

This study found that the nicotine, VG, and TSNA contents of IQOS sticks significantly differs between product categories (“Bold” vs. “Light”) and countries. Although no differences were found in the nicotine content and tobacco weight between products labeled “Bold” and “Light”, the “Bold” products overall contained higher levels of TSNAs. It is currently unknown whether the observed lower TSNA content in “Light” products could potentially result in reduced exposure to those toxic chemicals in people who use IQOS “Light” vs. those who use IQOS “Bold” products.

When comparing the concentrations of the analyzed chemicals in the IQOS products purchased from different countries, we found several significant differences. Specifically, significant differences in the IQOS stick TSNA concentrations were observed between countries, where products from the UK had lower NNN and NNK contents, while products from South Africa had elevated levels of NNN and NNK. Similar differences in NNK yields were previously reported for Marlboro cigarettes sold in the same countries [29]. Interestingly, the nicotine concentrations of all the sticks were consistent between countries, with a few exceptions driven by the overall lower concentrations in the IQOS products from Canada and the UK (Tables S2 and S3). These findings are inconsistent with previous studies, which report that nicotine yields among Marlboro tobacco cigarettes sold in various countries varied up to 83.8% between countries [29]. However, design features such as ventilation and puff topography can greatly affect nicotine yields, which do not affect nicotine content. One potential explanation for these significant differences by country is the sourcing of tobacco from various growth locations depending on where each pack is manufactured and sold. In addition, local curing methods may vary, resulting in differences in TSNA content.

We found that the amount of tobacco filler (tobacco weight) in the sticks remained uniform between countries. This suggests that the changes in the nicotine and TSNA content in the sticks are not driven by the amount of tobacco added but rather by the chemical composition of the tobacco filler. Due to the limited number of countries from the same regions (North America, Europe, Africa, and Asia), we did not analyze the differences between products purchased from each region. We have noticed, however, that many key chemical characteristics of IQOS sticks remained consistent between countries from the same region; for example, no significant differences were observed in the nicotine and TSNA content between the North American sticks (Canada vs. the US, as seen in Table S2).

Our study has confirmed the previously reported results that VG was the dominant humectant found in IQOS sticks [24,30]. In addition, our research also found that PG was still present in these products, as seen in combustible cigarettes [31]. Interestingly, the VG content in the sticks was significantly decreased in Japan and Poland compared to the other countries examined in this study. These changes are inversely proportional to the higher nicotine content in these products. As nicotine content regulation in various countries becomes enforced, varying the humectant content in the sticks may be a way for tobacco manufacturers to adjust the amount of nicotine delivered to the user.

A limitation of our study is that not all the sticks were purchased during the same period for all locations. The sampling period took place over three years and was determined by the availability of the researcher who collected these products. It is possible that the composition of sticks may have been modified by the industry over this time frame. Additionally, in several markets, the product brand names and the descriptors presented on the packaging have changed. In some countries, sticks designed for IQOS heating devices are no longer marketed under the name listed in this article (see Table S1). For example, in Canada, the “Silver” label product is now known as “Birch”, and the “Red” label is now known as “Elm”. This lack of consistent nomenclature of the IQOS stick flavors limits our understanding of these products, as it is unclear whether changes are only marketing tactics or whether chemical constituents such as nicotine, humectants, and TSNAs are also changing. Another limitation of our study is that products from both the “Bold” and “Light” categories were not purchased in each country represented. The authors made multiple attempts in each country to buy all the available IQOS sticks then; however, as this was a convenient sample, not all the samples listed for sale by PMI were purchased (see Table S1 for the current list). An additional limitation is that the purchasing location does not represent the location where the tobacco is grown or cured. As the source of tobacco can have a large impact on the composition of a tobacco product, knowing the source of tobacco would have resulted in more meaningful correlations as compared to by-country analysis. A final limitation is only one pack of sticks was purchased in each location. Additional packs would have allowed for further chemical analysis of these products, including emissions testing.

The chemical composition of IQOS sticks can vary significantly depending on the type (“Bold” vs. “Light”) and purchasing location. Our findings imply that the research related to IQOS sticks may vary depending on where the sticks are sourced globally. Researchers should consider these implications when designing future studies to estimate the effects of IQOS HTPs in their region. Future research related to IQOS sticks should also ensure that all products tested are sourced from the same “local” cohort.

## Data Availability

The data presented in this study are available on request from the corresponding author (accurately indicate status).

## Supplementary Materials

The following supporting information can be downloaded at https://www.mdpi.com/article/10.3390/toxics12030180/s1. Table S1: All IQOS stick varieties as of November 2023; Table S2: Additional analysis of “Light” IQOS sticks.; Table S3: Additional analysis of “Bold” IQOS sticks.
